# Characterization of Fatigue Damage in Hadfield Steel Using Acoustic Emission and Machine Learning-Based Methods

**DOI:** 10.3390/s24010275

**Published:** 2024-01-03

**Authors:** Shengrun Shi, Dengzun Yao, Guiyi Wu, Hui Chen, Shuyan Zhang

**Affiliations:** 1 Centre of Excellence for Advanced Materials, Dongguan 523808, China; guiyi.wu@ceamat.com (G.W.); shuyan.zhang@ceamat.com (S.Z.); 2 School of Materials Science and Engineering, Southwest Jiaotong University, Chengdu 610032, China; xnrpt@swjtu.edu.cn; 3 China Special Equipment Inspection & Research Institute, Beijing 100029, China; yaodengzun@csei.org.cn

**Keywords:** crack growth, acoustic emission, machine learning, Hadfield steel

## Abstract

Structural health monitoring (SHM) of fatigue cracks is essential for ensuring the safe operation of engineering equipment. The acoustic emission (AE) technique is one of the SHM techniques that is capable of monitoring fatigue-crack growth (FCG) in real time. In this study, fatigue-damage evolution of Hadfield steel was characterized using acoustic emission (AE) and machine learning-based methods. The AE signals generated from the entire fatigue-load process were acquired and correlated with fatigue-damage evolution. The AE-source mechanisms were discussed based on waveform characteristics and bispectrum analysis. Moreover, multiple machine learning algorithms were used to classify fatigue sub-stages, and the results show the effectiveness of classification of fatigue sub-stages using machine learning algorithms. The novelty of this research lies in the use of machine learning algorithms for the classification of fatigue sub-stages, unlike the existing methodology, which requires prior knowledge of AE-loading history and calculation of ∆K.

## 1. Introduction

Metal fatigue is a phenomenon of local cracks or global fracture of components under cyclic loading, and it accounts for 70–80% of structural accidents across different types of engineering equipment [1]. Traditionally, non-destructive testing techniques have been employed to inspect engineering components periodically to ensure safe operation and predict the remaining useful lifespan. With efficient non-destructive testing (NDT), it is possible to detect fatigue damage at even initial stages, thus avoiding immense economic and life losses. However, critical fatigue damage may occur between the times at which NDT is normally performed. Recently, there has been increasing interest in adopting structure-health monitoring (SHM) technology for the evaluation and monitoring of engineering components through the use of sensors permanently installed on the structures. One main advantage of SHM over conventional NDT techniques is that it is not performed periodically; therefore, the possibility of fatigue failure between tests is excluded [2]. SHM can be considered a typical inverse problem, which is often ill-conditioned, because the relationship between measured data (such as sensor readings) and structural health parameters (such as damage or condition) is often complex, non-linear, and sensitive to measurement noise. In view of this, researchers have recently employed various approaches, including novel regularization methods, sensing methods, etc., to address problems related to inverse structural dynamics [3,4].

The fatigue-degradation process can be classified into three stages based on the logarithmic relationship between the crack-growth rate (da/dN) and the stress-intensity factor range (ΔK); namely, stage 1 for initial slow growth, stage 2 for stable linear growth, and stage 3 for final rapid growth. In stage 1, a crack initiates when ΔK reaches the threshold value ($K_{th}$) and then it grows slowly along primary slip planes. In stage 2, the crack-growth rate follows the Paris–Erdogan law, in which the relationship between da/dN and ΔK is approximately linear on a double-logarithmic scale. In stage 3, the crack becomes unstable and grows rapidly until final failure. Previous studies have reported the transition behavior in fatigue-crack growth rate (FCGR) curves in stage 2 for various kinds of metallic materials [5,6,7]. Specifically, stage 2 can be divided into two sub-stages, namely stage 2a and stage 2b, by a transition point. The transition point is material-dependent and may be associated with a transition of the governing micro-mechanisms of crack growth. During stage 2a, da/dN depends strongly on the microstructure and the crack does not grow in every cycle. When crack growth enters into stage 2b, da/dN becomes insensitive to the microstructure and the crack grows stably with fatigue striations appearing on the fracture surfaces. However, it has been pointed out by some authors [8,9,10] that it is difficult to discern these two sub-stages through mechanical testing data and that overlapping of these two sub-stages sometimes makes them indistinguishable. Notwithstanding this challenge, it should be noted that the recognition of the two sub-stages can be beneficial for the accurate assessment of fatigue damage and remaining fatigue lifespan, which will reduce the risk of unstable crack growth and give early warning of failure. Therefore, the accurate identification of the two sub-stages is of great significance. 

Acoustic emission (AE) is widely used as an SHM technique for monitoring damage evolution in metallic structures. The technique is based on the detection of elastic stress waves emitted during damage evolution while a structure is loaded. Fatigue-crack growth (FCG), such as any sudden local change of irreversible strain, is a typical AE source. Indeed, the continuous monitoring of the FCG process is one of the most important applications of AE, which is difficult to be achieve using any other non-destructive evaluation techniques. Investigations have been conducted extensively to establish the correlation between fatigue damage and AE characteristics [11,12,13,14,15,16,17]. For instance, Aggelis et al. [11] presented a method using certain AE parameters to characterize the damage evolution and the change in fracture mode in metal plates. Specifically, the RA (ratio of rise time to amplitude) value, duration and risetime showed a rapid increase prior to the final fracture, indicating the shift from the tensile to the shear fracture modes. Chai et al. [14] and Han et al. [12] divided fatigue process into three stages based on the evolution of time-domain AE parameters, namely stage 1 for crack initiation, stage 2 for stable crack growth, and stage 3 for rapid crack growth and fracture. Moreover, it is widely accepted that an approximately log-linear relationship between count rate and ΔK exists by only collecting AE events occurring near the peak load, assuming that these AE events are directly caused by crack extension [16,17]. However, some authors have pointed out that for ductile materials, continuous AE-signal collection during entire fatigue cycle representing the complete damage can obtain further important information on the FCG process from all possible signal sources, which is otherwise difficult based on AE-signal collection only near the peak load [18,19,20]. 

Despite the vast amount of study on the characterization of fatigue damage using acoustic emissions, only a few studies have investigated the relationship between the two sub-stages during FCG and the AE activity. It has been proved that the evolution of the cumulative count with ΔK plot can be used to identify the transition from stage 2a to stage 2b. For instance, Chai et al. [10] found that the transition from stage 2a to stage 2b of the FCG process in 316LN stainless steel and welds is accompanied by a slope change in the AE cumulative count vs. ΔK plot. The observed transition point based on AE activity is consistent with the theoretically calculated value. For in-service structure, identification of two sub-stages is still a challenging task due to the following reasons. Firstly, the exact load-time information required for ΔK calculation for in-service structures may not be easily obtained [21]. Secondly, it is extremely hard to derive the stress-intensity factor in an analytical manner for in-service structures with complex geometries [22]. Thirdly, AE-signal history, which is required prior to cumulative count calculation, may not also be available [23]. Therefore, more robust data interpretation is needed for the identification of the two sub-stages. In this matter, advancements in computer algorithms and hardware have made the implementation of machine learning techniques for damage characterization more feasible. In the field of SHM, machine learning has the potential to deal with complex problems related to non-linear relationships and can help to improve prediction accuracy [24]. As such, many researchers started using a machine learning approach for the health monitoring of various structural systems in the last decade [23,25,26,27,28]. For instance, Sikdar et al. [25] proposed a convolutional neural network (CNN)-based approach for identification of the region of damage in a composite panel. Garret et al. [23] utilized a combination of Choi–Williams transform (CWT) and convolutional neural network to predict fatigue-crack length in aluminum plates. Chai et al. [28] developed a back propagation neural network optimized by genetic algorithm (GA-BPNN) for FCGR prediction based on AE monitoring data. It is noticed from the literature that despite the significance of identification of fatigue sub-stages, research is still missing that uses machine learning-based methods to identify fatigue sub-stages. In view of this, the main objective of this study is to investigate AE behaviors during the FCG process in Hadfield steel and to investigate the possibility of using machine learning algorithms for the classification of fatigue sub-stages. The novelty of this research lies in the development of an automated approach for classification of the two sub-stages based on machine learning algorithms using AE signals, unlike the existing methodology, which requires prior knowledge of AE-loading history and calculation of ∆K.

This article is organized as follows: Section 2 describes the experimental setup and procedures and briefly introduces the algorithms used for classification of fatigue sub-stages. Section 3 discusses the experimental results. Section 4 presents the conclusions of this article.

## 2. Methodology

### 2.1. Materials and Specimens

**Figure 1:**
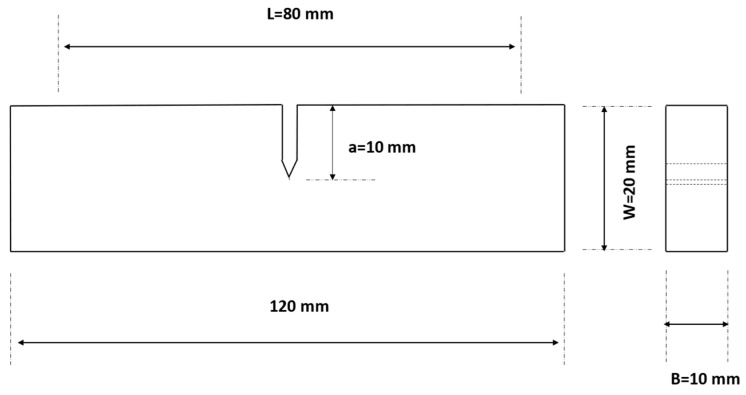
Schematic diagram showing the specimens used for fatigue-crack growth tests.

Standard single-edge notched specimens were extracted from a Hadfield steel section made of UIC 866 grade (supplied by West Yorkshire Steel Ltd., Leeds, UK, 1.2 C and 11–14 Mn in wt%) with dimensions 120 mm × 20 mm × 10 mm. The schematic diagram of the specimens is shown in Figure 1 and the nominal mechanical properties of Hadfield steel are given in Table 1.

### 2.2. Test Instrument and Procedure

Three-point bending fatigue tests were carried out on a Dartec 50 kN servo-hydraulic universal test machine at ambient temperature (300 K). Fatigue-crack growth tests were carried out on two pre-cracked Hadfield steel specimens, namely specimen 1 and specimen 2, and on a dummy specimen with no known cracks present. All specimens were tested under sinusoidal-cyclic loading at a frequency of 1 Hz and load ratio of 0.1. The distance between the two support rollers was 80 mm. The maximum load was set at 3 kN. The pre-cracked lengths for specimen 1 and specimen 2 are 10.2 cm and 10.4 cm, respectively. The crack length during FCG was monitored using the Direct Current Potential Drop (DCPD) technique. DCPD is based on the principle that the resistance of the material will increase with the decreasing size of cross section resulting from a fatigue crack growing. Constant direct current (DC) was input through two copper clips into the specimens and the voltmeter measured the voltage drop across the notch. The crack-length data and ΔK were calculated after tests. The fatigue-crack growth rate was calculated using a 5-point secant method (ASTM E647-15e1). The equation used for calculating ΔK is shown below [30]:

(1) $$\Delta K=\frac{6\Delta P}{B\sqrt{W}}a^{1/2}Y$$

where ΔP—load range; a—crack length; B—thickness of the specimen; W—width of the specimen. Y is dimensionless function dependent on the loading configuration and geometry of the specimen and it is calculated as below [30],

(2) $$\;Y=\frac{1.99-\left(\frac{a}{W}\right)\left(1-\left(\frac{a}{W}\right)\right)\left(2.15-3.93\left(\frac{a}{W}\right)+2.7{\left(\frac{a}{W}\right)}^{2}\right)}{\left(1+2\left(\frac{a}{W}\right)\right){\left(1-\left(\frac{a}{W}\right)\right)}^{\frac{3}{2}}}.$$

### 2.3. Acoustic Emission Monitoring 

**Figure 2:**
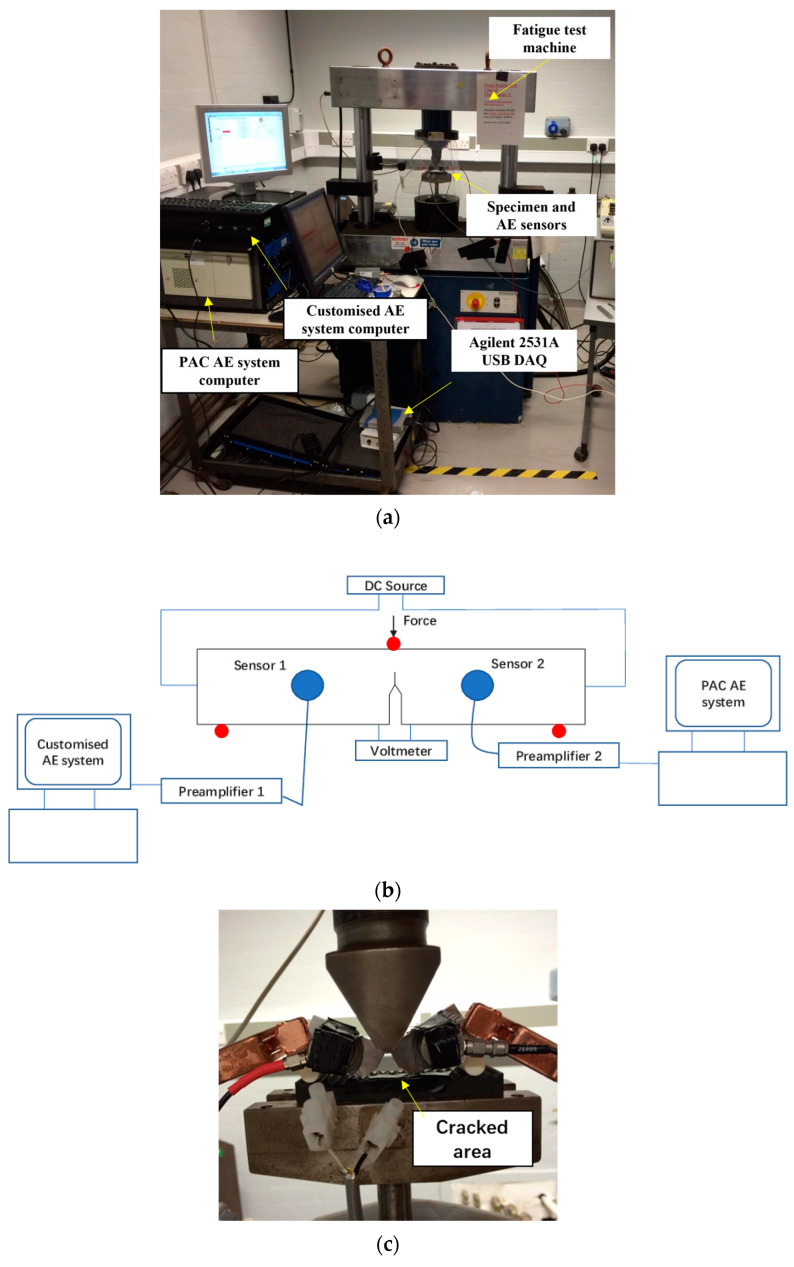
(**a**) Photograph of the experimental setup, (**b**) schematic diagram of the experimental setup, and (**c**) photograph showing the specimen after the final failure occurred.

The customized AE system developed by the researchers from the University of Birmingham and Krestos Limited and the commercial AE system procured from Physical Acoustic Corporation (PAC) were employed to capture the AE signals generated during the tests. In the customized system, an Agilent 2531A 2 MSamples/s data acquisition (DAQ) card was used to capture AE signals whereas the logging and signal processing software was written in Matlab R2014b. For the customized system, a five-second-long AE measurement was taken every 5 min and then as the testing progressed, the measurement was taken at 1-min intervals. The sampling rate was set at 1 MSamples/s during the tests. Two R50a sensors produced by PAC were attached to the surface using grease and were kept in place with adhesive tapes. The R50a sensor was chosen mainly due to the fact that it is well-suited for testing in environments where high levels of external noise generate low-frequency signals that must be filtered out during measurement. The available parameters of the R50a sensor are shown in Table 2. During the test, one R50a sensor was connected to the PAC system and the other one was connected to the customized system. The AE sensors were mounted approximately 20 mm away from the center of the specimen, one on either side of the cracked region. The sensors were connected to preamplifiers set at 40 dB gain. The hit-defining parameters used in AE testing, including peak-definition time, hit-definition time and hit-lockout time were set at 300, 600, and 1000, respectively. To eliminate the interference of external noise during testing, both amplitude threshold and band-pass filter were used in the PAC system. Specifically, the AE amplitude threshold during recording was set as 45 dB to minimize the number of mechanical noise-related hits being recorded during tests. Moreover, an analogue 20 kHz high-pass filter was used to remove low-frequency background noise. A photograph and schematic diagram of the experimental setup is shown in Figure 2.

### 2.4. Machine Learning Algorithms

Three machine learning algorithms were used in this study: Multilayer Perceptron (MLP), k-Nearest Neighbor (kNN), and Support Vector Machine (SVM). The principle of each algorithm is briefly introduced below.

#### 2.4.1. Multilayer Perceptron

The MLP is a feedforward artificial neural network, which has an input layer, one or more hidden layers, and an output layer [24]. The input layer has as many neurons as the input features and transfers the data to the hidden layer to be processed. The hidden layer is basically an intermediate layer between the input and the output layer of the network. The output layer has as many neurons as the number of output variables. Each neuron in the hidden and output layers consists of an activation function φ, which is generally a non-linear function. The non-linear activation function converts linear input to non-linear outputs and usually limits the outputs to values between 0 to 1, or −1 to +1.

(3) $$y=\varphi (\sum_{i=1}W_{ij}\varepsilon_{i}+b)$$

where y is the output of the neuron, φ is the activation function, $W_{ij}$ is the weight associated with the connection between input $\varepsilon_{i}$ and the neuron’s output, $\varepsilon_{i}$ is the input from the i-th neuron, and b is the bias. In this study, a backpropagation algorithm was used to update the network weights until the convergence criteria was met. Detailed information about the backpropagation algorithm can be found in [31].

#### 2.4.2. k-Nearest Neighbor

kNN is a non-parametric, supervised learning algorithm. The idea of this method is to allocate new unclassified data points in the class to which the majority of its k-nearest neighbors belongs. The classification accuracy of the kNN algorithm is mainly dependent on the value of k and the type of distance metrics used for calculating the nearest distance. Commonly used distance metrics include Euclidean distance, Manhattan distance, or cosine similarity [32]. In this study, Euclidean distance was used as the distance metric. 

#### 2.4.3. Support Vector Machine

SVM is a supervised learning method that aims to find the hyperplane with the maximum margin that allows the samples to be maximally separated [33]. For a given training set, $D={\left\{x_{k},y_{k}\right\}}_{k=1}^{N}$ with $x_{k}\in R^{n}$ being the feature vectors and $y_{k}\in${−1, +1} is the class label, and the corresponding hyperplane equation is as follows,

(4) $$f\left(x\right)=w^{T}\varphi \left(x_{k}\right)+b$$

where $f\left(x\right)$ is the decision function, $w^{T}$ is the transpose of the weight vector, φ is the kernel function, $x_{k}$ is the feature vector and b is the bias. The feature vector is mapped to the high-dimensional feature space to make data points linearly separable using the kernel function φ and the objective is to minimize the following function,

(5) $$\frac{1}{2}{\left\|w\right\|}^{2}+\lambda \sum_{k=1}^{N}\xi_{k}$$

under the following constraints,

(6) $$y_{k}\left[w^{T}\varphi \left(x_{k}\right)+b\right]\geq 1-\xi_{k}\;;\;k=1,\ldots ,N$$

(7) $$\xi_{k}\geq 0;k=1,\ldots ,N$$

where $y_{k}$ is the class label, w is the weight vector, λ is the penalty parameter and $\xi_{k}$ is the slack variable.

In the present study, the above optimization problem was solved by using the Lagrangian Method. Moreover, the radial basis function (RBF) kernel was used as the kernel function. 

### 2.5. Feature Selection and Model Training 

The selection of AE parameters is crucial for accurately assessing fatigue-damage condition and classifying fatigue sub-stages. Only by utilizing more suitable AE parameters can better results be obtained. Table 3 shows the AE features (time-domain and frequency-domain) used to train the algorithms. In this study, time-domain AE features were used as part of the input features, as they tend to directly reflect the severity of fatigue damage. Among the time-domain features, amplitude, count, energy, duration, and risetime are fundamental parameters of AE, and they play a more significant role in the analysis since other parameters can be directly or indirectly inferred from them. However, the use of time-domain features alone is not sufficient, as they can be subjected to the influence of sensor location, acquisition setting, etc. Therefore, frequency-domain features such as bispectral-based features, frequency centroid, and peak frequency were also included in the input features, since they are less affected by testing condition such as specimen, sensor location, etc. [34]. Moreover, as one of the higher-order spectra, the bispectrum can suppress gaussian noise and increase the signal-to-noise ratio of signals [35]. In this study, bispectral-based parameters were obtained after performing singular value decomposition (SVD) of bispectrum matrix and the features, including maximum singular value, average singular value, and variance of singular value were extracted. SVD has been widely used as a feature extraction tool and can reduce the quantity of data while still retaining the important information [36]. According to the SVD theorem, the bispectrum matrix A ∈ Rm × n can be decomposed into the product of matrices as shown below,

(8) $$A=U\sum V^{T}$$

where U is the orthogonal m × m unitary matrix, V is the orthogonal n × n unitary matrix, and ∑ (the same dimensions as A) has the singular values and is diagonal. 

After feature extraction, AE signals were labelled according to the sub-stages they belonged to and the corresponding dataset was divided into training and testing sets with a 7:3 ratio. The RandomizedSearchCV (i.e., random search and 5-fold cross-validation) method was used to select the optimal model hyperparameters, which is an automated operation for tuning parameters. The machine learning libraries Pandas, NumPy and Sklearn (i.e., Scikit-learn) in Python were used in the development of these algorithms. 

## 3. Results and Discussion

### 3.1. Characteristics of AE during FCG

#### 3.1.1. Overall Fatigue and AE Behaviors

**Figure 3:**
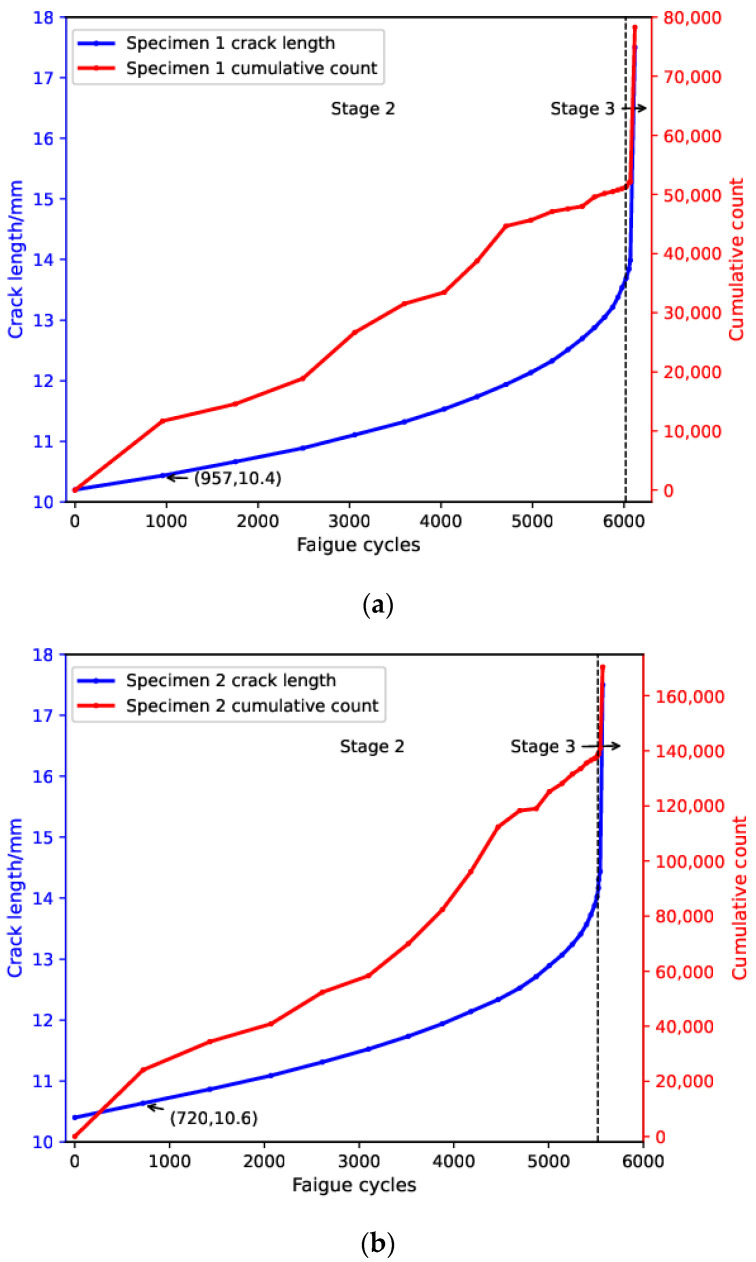
Evolution of crack length and cumulative AE count with fatigue cycles for (**a**) specimen 1 and (**b**) specimen 2.

Figure 3 shows the evolution of crack length and cumulative AE count with fatigue cycle for both specimens. The crack lengths when the specimens failed were around 17.5 mm in both cases. Based on crack-length evolution, the fatigue test was divided into two stages, namely stable crack-growth stage (stage 2) and rapid crack-growth stage (stage 3). Moreover, the fatigue lifespan was dominated by stage 2 during which crack length increased approximately linearly. During stage 3, crack length increased rapidly until final failure. Cumulative AE count showed a similar trend with crack length during the test. Specifically, the cumulative count exhibited a quasi-linear growth until around 6000 cycles and 5500 cycles for specimens 1 and 2, respectively, and showed a sudden increase for both specimens at stage 3, indicating imminent final failure. It is also noteworthy that AE counts were accumulated in a relatively rapid manner from the beginning until 957 cycles and 720 cycles, respectively, and this indicated the influence of an additional source mechanism. Given the fact that crack lengths in both specimens increased around 0.2 mm during this time period, this additional source mechanism can be attributed to residual stress relaxation in the plastic zone ahead of the crack tip induced in the fatigue-pre-cracking process, as compressive residual stresses were created in the vicinity of the crack tip as a consequence of the formation of a cyclic plastic zone during the fatigue-pre-cracking process [37].

#### 3.1.2. Identification of Fatigue Sub-Stages and the Corresponding AE Characteristics

**Figure 4:**
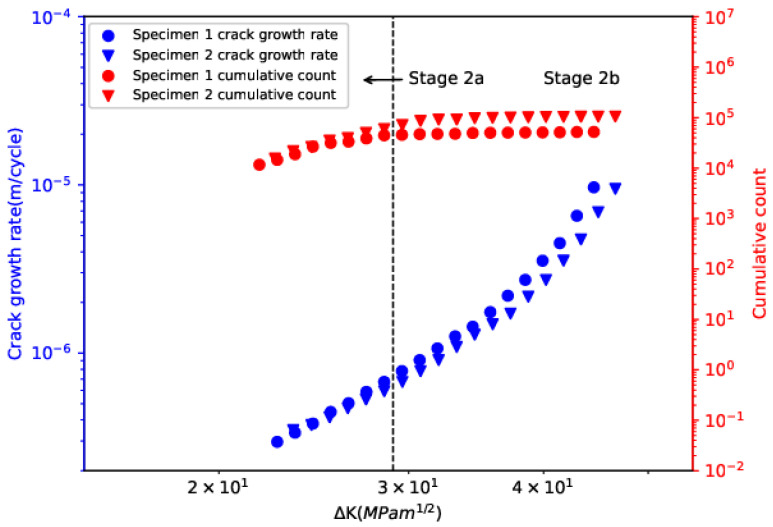
Evolution of crack-growth rate and cumulative count with ∆K.

Figure 4 illustrates the evolution of crack-growth rate and cumulative count with ∆K. Cumulative count exhibited obvious transition behavior and two sub-stages, namely stage 2a and stage 2b, can be clearly visualized. The ∆K at the transition between the two sub-stages $\left(∆K_{0}\right)$ was found to be around 28.5 MPa·m^1/2^. The transition can be attributed to the change in stress state from plane-strain dominated to plane-stress dominant [8]. The empirical equation for calculating $∆K_{0}\;$ given by the literature is as follows [38,39],

(9) $$∆K_{0}=10*(Eb^{1/2})$$

where E is Young’s modulus and b is Burger’s vector. According to the above equation, $\Delta K_{0}$ was calculated as 29.4 MPa·m^1/2^, using the value of E as 186 MPa and b as 2.5 × 10^−10^ m [40]. Hence, the experimentally observed transition value was close to that calculated from the empirical equation.

**Figure 5:**
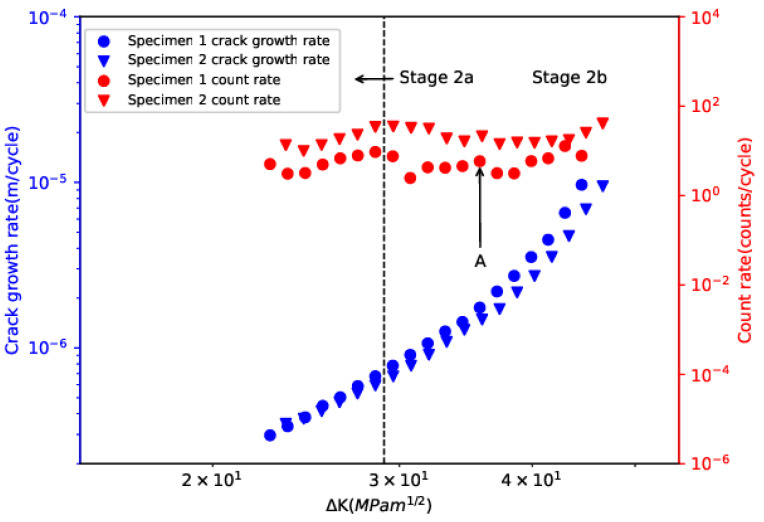
Evolution of crack-growth rate and count rate with ∆K.

Figure 5 shows crack-growth rates (da/dN) and AE count rates (dC/dN), calculated using a 5-point secant method, versus ΔK on the double-logarithmic axes. The relationship between da/dN and ΔK was described based on the Paris–Erdogan law for both specimens [41]:

(10) $$\frac{da}{dN}=C\Delta K^{m},\;\mathrm{or}:\;\log(\frac{da}{dN})=\log C+m\log\Delta K$$

where C and m are assumed to be constants for a particular material. The relationship between dC/dN and ΔK was described similarly as [42,43]:

(11) $$\frac{dC}{dN}=B\Delta K^{p},\;\mathrm{or}:\;\log(\frac{dC}{dN})=\log B+p\log\Delta K$$

where B and p are constants for a particular material and loading condition. The fitting values of parameters for da/dN and dC/dN are summarized in Table 4. Based on R-squared values, da/dN and ΔK showed very strong correlation, whereas the correlation between dC/dN and ΔK was weak. The weak correlation between dC/dN and ΔK is expected due to the obvious variations observed in dC/dN. Specifically, during stage 2a, dC/dN showed an upward trend and reached a peak value near the transition point. During stage 2b, instead of a continuous increase with ΔK, dC/dN exhibited more obvious discontinuities and two sub-regions, namely region 1 from 28.5 Mpa·m^1/2^ to 35.7 Mpa·m^1/2,^ and region 2 from 35.7 Mpa·m^1/2^ to 46.6 Mpa·m^1/2^, can be identified by the “A” marking in Figure 5. At the end of region 2, there was a sharp increase in dC/dN, indicating the transition from stage 2b to stage 3. The mechanisms for the obvious discontinuities observed during stage 2b will be discussed later. Previous studies [17,42] have proposed the use of dC/dN to predict da/dN during the FCG process with the approximation that the relationship between dC/dN and ΔK is similar to the Paris–Erdogan law. However, the present results indicate that prediction of da/dN using dC/dN based on the relationship being similar to the Paris–Erdogan law may not be reliable for the investigated material due to the obvious discontinuities exhibited by AE behavior. 

**Figure 6:**
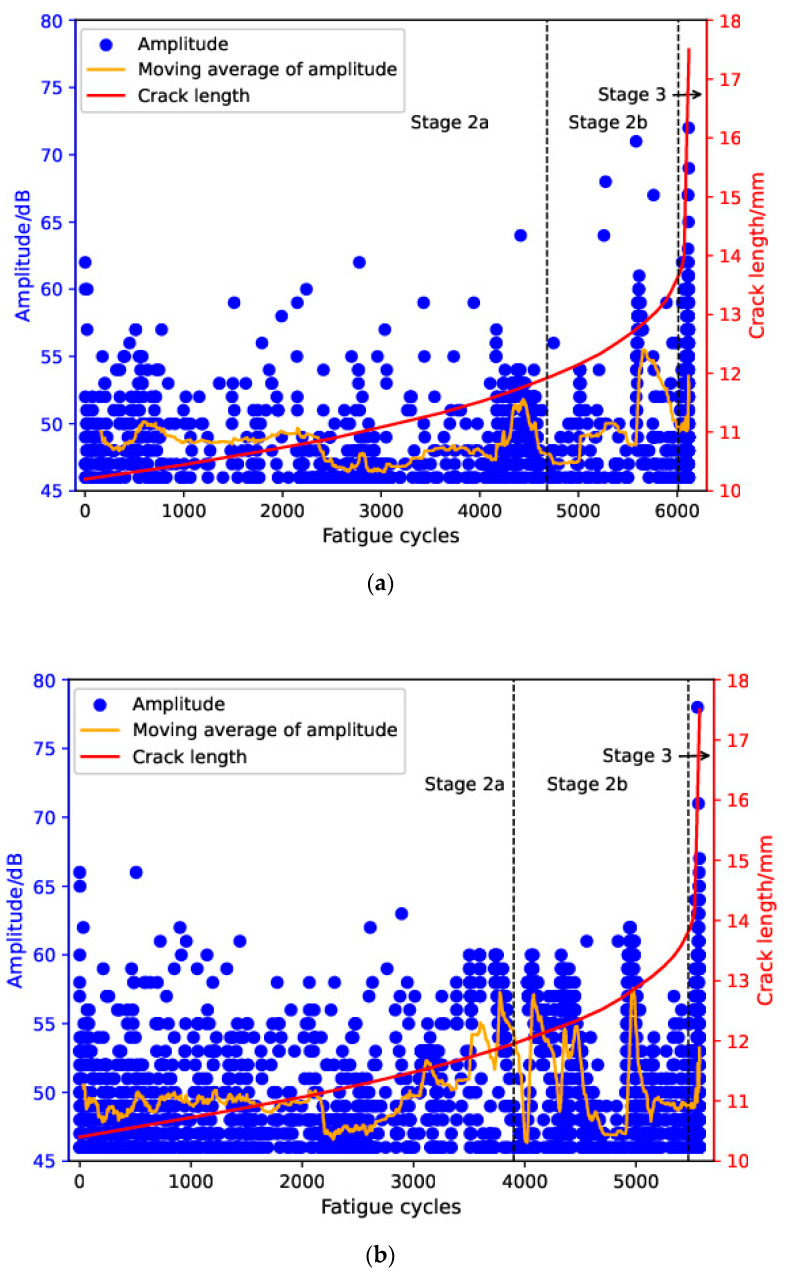
Evolution of amplitude and crack length with fatigue cycles for (**a**) specimen 1 and (**b**) specimen 2.

Figure 6 shows the plot of crack length versus fatigue cycles with the corresponding AE amplitude for both specimens. The moving average of the amplitude values of the most-recent 150 AE hits was also included in the figure for comparison. Amplitude is often used for characterization of the time–history of AE activity during fatigue failure of material and it is sensitive to source mechanisms. Low stacking-fault energy (SFE) of the austenitic structure in Hadfield steel makes high-amplitude (>=70 dB) AE events related to brittle fracture less likely to occur during stable crack propagation, which has also been confirmed by the current results [44,45]. Specifically, most AE events during stable crack propagation are distributed in the range of 45 to 60 dB. For both specimens, near the end of stage 2a, a cluster of AE signals with amplitudes above 50 dB appeared and the moving average increased sharply. However, it should be noted that accurate identification of the sub-stages based on amplitude and its moving average is hardly achievable, given the obvious fluctuation they exhibited during the tests.

#### 3.1.3. Identification of AE Mechanisms Based on Waveform Characteristics

**Figure 7:**
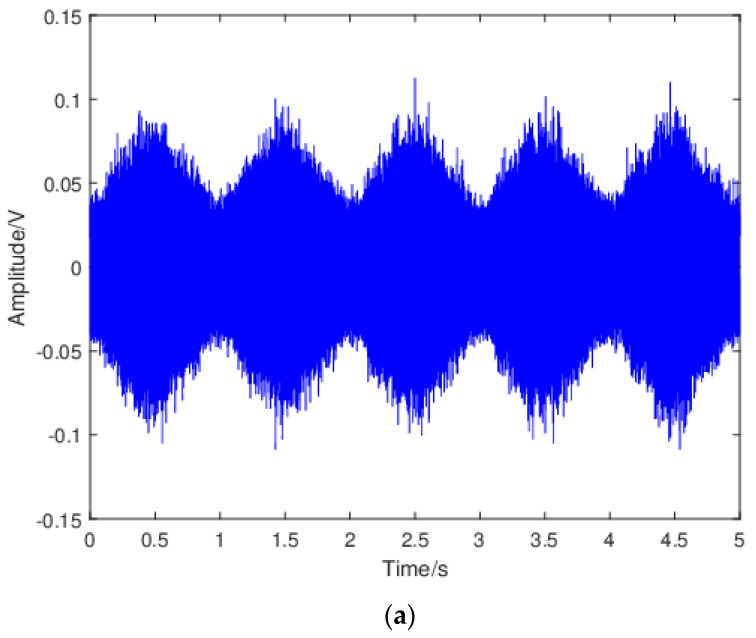

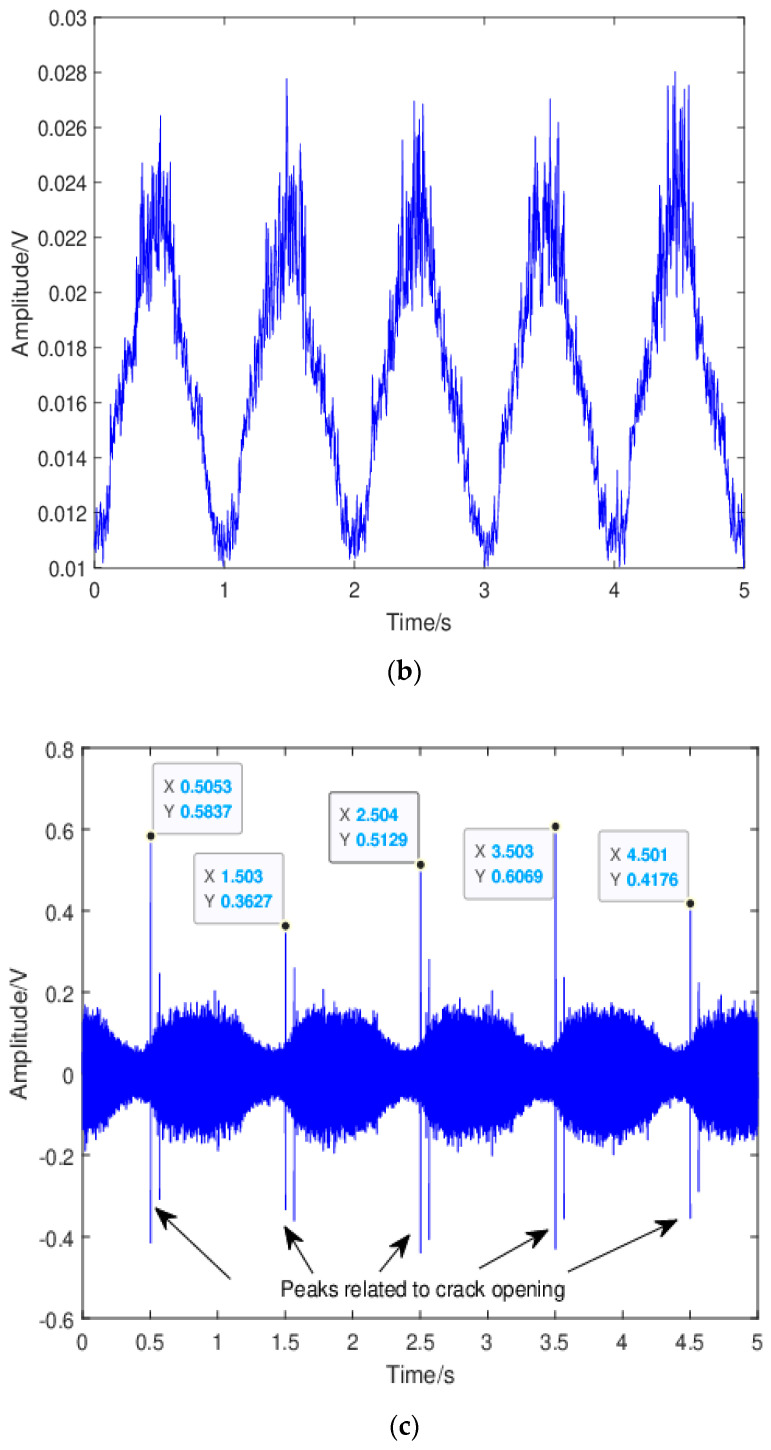

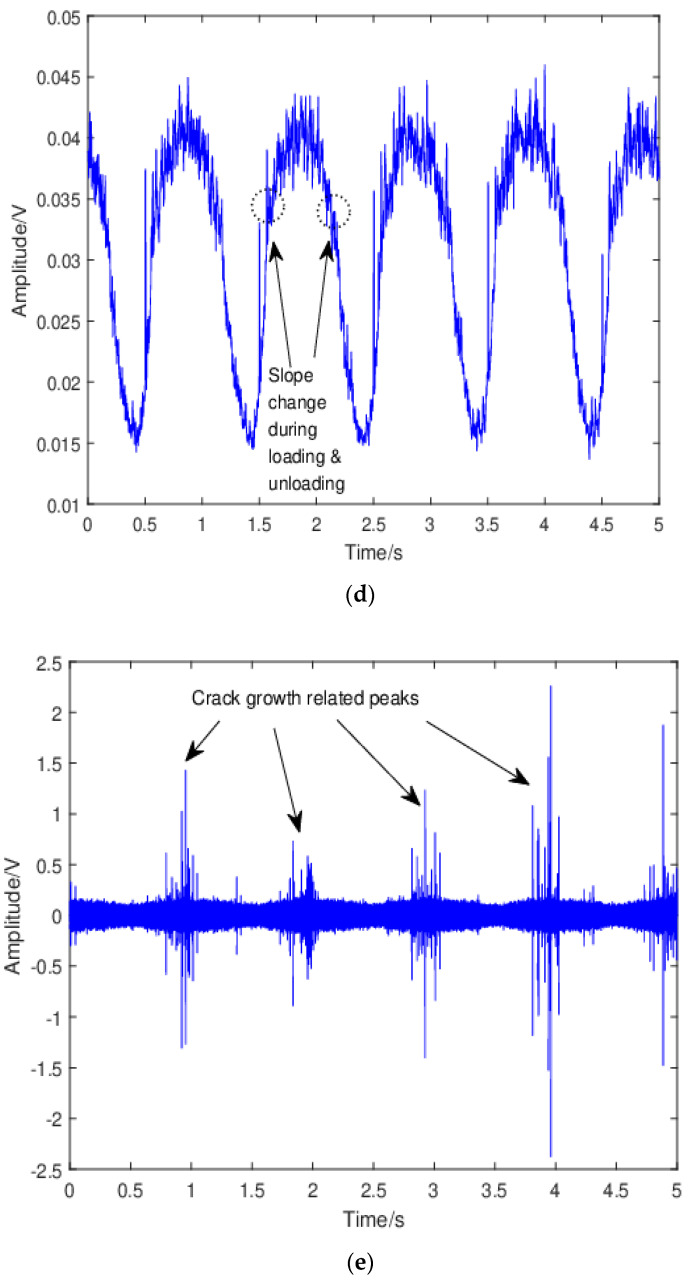

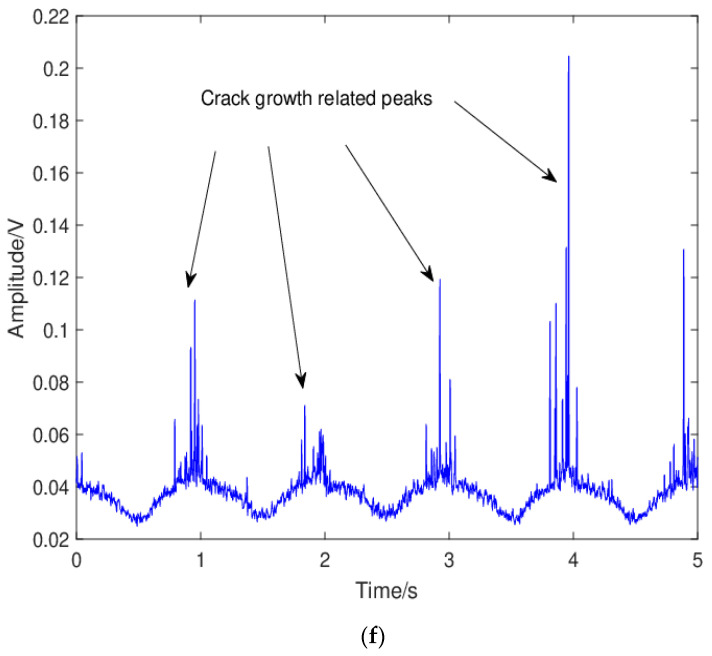
The representative waveform for (**a**) the dummy specimen, (**c**) specimen 2−region 1 of stage 2b, (**e**) and specimen 2−region 2 of stage 2b; the corresponding moving RMS for (**b**) the dummy specimen, (**d**) specimen 2−region 1 of stage 2b, and (**f**) specimen 2−region 2 of stage 2b.

To gain insight into the mechanisms responsible for the observed discontinuities during stage 2b, the raw waveforms were investigated. Figure 7 shows the representative waveforms and the corresponding moving RMS values during the 5-s acquisition window for region 1 and 2 of stage 2b for specimen 2. The waveform for the dummy specimen was also included for comparison purposes. The moving RMS was calculated using a window size of 5000 with 75% overlapping. For the dummy specimen, the sinusoidal-cyclic-loading pattern was clearly observed in the waveform. This was expected since no crack growth occurred in the dummy specimen. Moreover, the moving RMS changed in a quasi-linear fashion during loading and unloading in each fatigue cycle. For the waveform in region 1, clear repetitive peaks occurring every 1 s on the rising part of the waveform as shown by the datatips that were observed, and they can be attributed to crack opening (“unsticking” process) as reported in [6]. The occurrence of crack opening/closing was in agreement with the widely accepted fact that the plasticity-induced crack closure (PICC) is much more pronounced under a plane-stress than a plane-strain condition, resulting in a higher percentage of load range during which the crack is closed within single loading cycle. In the moving RMS plot, the peaks related to crack opening were suppressed. Moreover, slope change during loading and unloading within each fatigue cycle was observed and this can be attributed to the change in the dominant source mechanisms. Specifically, during loading, the dominant source mechanism changed from contact or rubbing between the crack surfaces to crack growth when crack was fully open, whereas when the crack started to close, contact or rubbing between the crack surfaces became the dominant source mechanism. The results also showed that moving RMS for the crack-growth-related signal was higher than that for the crack surfaces rubbing/contact related signal, which suggested that the crack opening/closing phenomenon can be differentiated from crack growth using AE moving RMS. Yusof et al. [46] investigated AE characteristics during fatigue of an API 5L X70 gas pipeline steel and found that AE signals related to crack closure had higher count values than those related to crack growth. However, AE RMS from crack propagation was higher than that from crack closure. These results are consistent with the present study. 

For the waveform in region 2, several crack-growth-related peaks can be observed in each loading cycle, indicating intensified crack-growth activity. Crack-growth-related peaks were also observed in the moving RMS plot. Notice that no obviously repetitive peaks can be observed in this case, indicating no crack opening/closing. This is reasonable, since the influence of the crack closing- and opening-effects decreases with increasing of the crack length or ΔK until little or no closure occurs in the case of 0 ≤ *R* < 0.5 [43]. The gradually intensified crack-growth activity contributed to the overall increasing trend in count rate in region 2.

#### 3.1.4. Bispectrum Analysis

**Figure 8:**
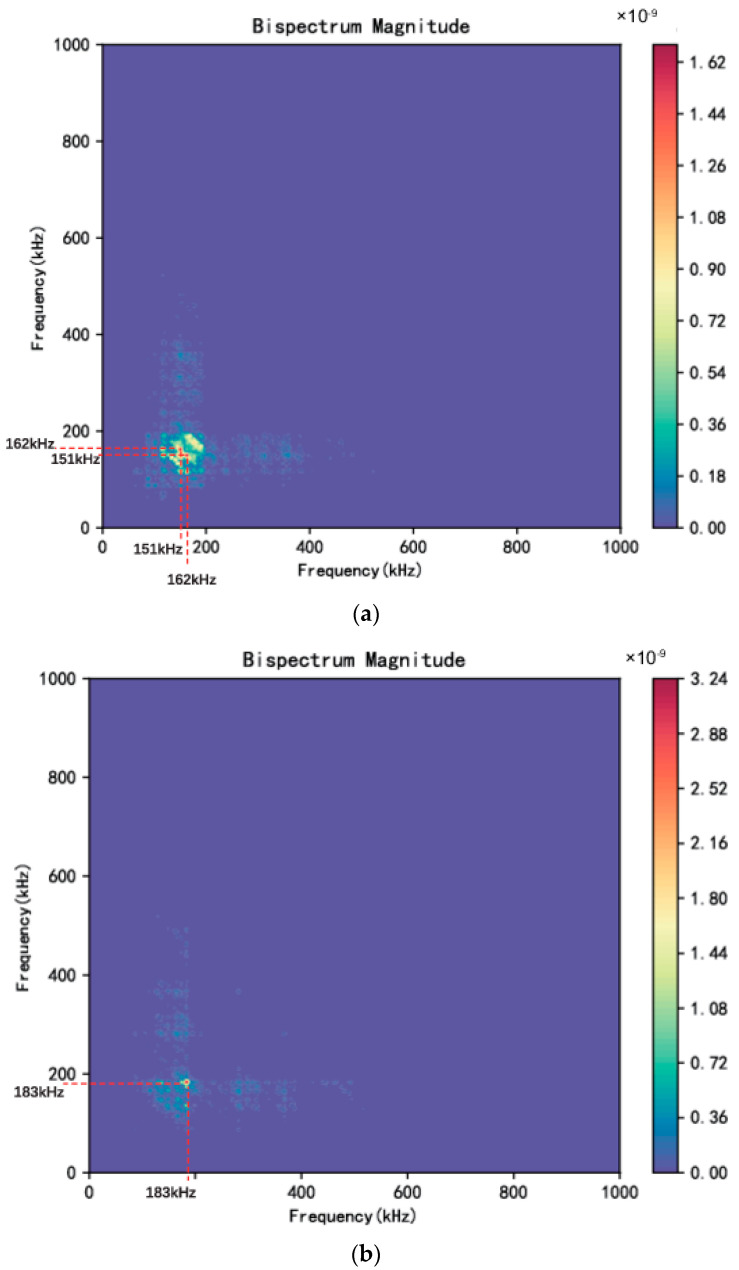
Contour plots of the bispectrum magnitudes for (**a**) ‘Type I’ (**b**) ‘Type II’ bispectrum sub-types.

Bispectrum is the most widely used higher-order spectrum and can provide the third-order statistic information of the signal. Bispectrum has been proved to be effective in detecting non-gaussian signals in the presence of disturbing noise [35]. It can be calculated by indirect methods such as Fourier transform of the third-order cumulant. The third-order cumulant $C_{3x}(k,j)$ and the corresponding bispectrum $B\left(f_{1},f_{2}\right)$ at frequencies f_1_ and f_2_ is defined as below,

(12) $$C_{3x}(k,j)=E\left\{x\left(t\right)\right.x\left(t+k\right)\left.x(t+j)\right\}$$

(13) $$B\left(f_{1},f_{2}\right)=\int_{-\infty }^{+\infty }\int_{-\infty }^{+\infty }C_{3x}(k,j)e^{-j(f_{1}k+f_{2}k)}dkdj$$

where $E\left\{\cdot \right\}$ is the statistical mean and k, j are time lags. In this study, bispectrum analysis was performed using the Python package Stingray. Based on contour plots of the magnitude of bispectrum during FCG, they could be predominantly grouped into two types, namely, a ‘Type I’ and ‘Type II’ bispectrum as shown in Figure 8. As can be seen, the magnitude distribution of the ‘Type I’ bispectrum is relatively dispersed and for this specific signal the peaks appear at (162 kHz, 150 kHz) and (150 kHz, 162 kHz), whereas the ‘Type II’ bispectrum exhibits a more concentrated magnitude distribution with the peak appearing at (183 kHz, 183 kHz) and its peak magnitude is higher than that for ‘Type I’. The peak at (183 kHz, 183 kHz) clearly indicates the existence of harmonics in the AE waveform, which is a manifestation of high acoustic non-linearity in the specimen. This difference in the magnitude distribution between the two types of bispectrum could be associated with different degrees of non-linearity due to the change in stiffness value of the specimen with an opened vs. closed crack. It is generally accepted that a crack in a metallic structure causes the non-linear interaction of acoustic waves, and the non-linear effects are closely related to the stiffness of the structure. Specifically, non-linear effects are more pronounced in specimens with higher stiffness than those with lower stiffness [47,48,49]. In the present study, when crack closure occurs, the structural stiffness increases, as the crack length of the opened crack is decreased. Therefore, a ‘Type I’ bispectrum should be related to crack growth, whereas a ‘Type II’ bispectrum should be related to crack closure. Differentiation between crack growth and crack closure is important and useful from the point of view of providing evidence about the sub-stage of crack growth, as crack closure is more pronounced when a plane-stress condition is dominant at stage 2b.

### 3.2. AE Signals Classification Results

**Figure 9:**
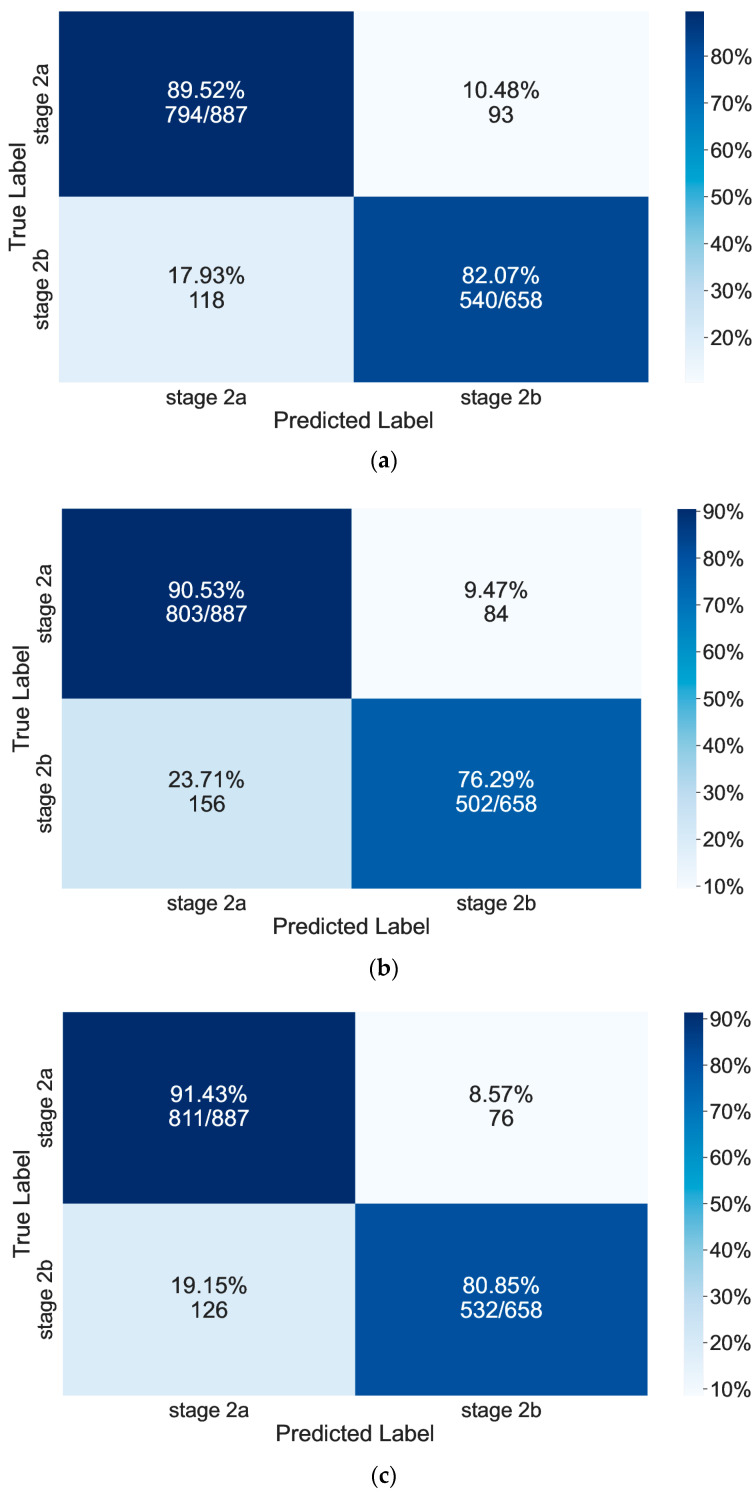
Confusion matrix showing the accuracy of (**a**) MLP, (**b**) kNN, and (**c**) SVM classifiers.

When evaluating the robustness of the method employed for data classification, model accuracy is a crucial factor that needs to be considered [50,51,52]. The accuracy value was determined through comparing the actual and predicted results. Those values were visually represented using a confusion matrix and classification metrics. The results from the classification are presented in the form of a normalized confusion matrix and classification metrics as shown in Figure 9 and Table 5, Table 6 and Table 7.

The accuracy of the MLP algorithm on the test dataset was 86%. Specifically, among the signals in stage 2a, 89.52% of them were correctly classified, while 17.93% of them were misclassified as signals in stage 2b. Among the signals in stage 2b, 82.07% of them were correctly classified, while 10.48% of them were misclassified as signals in stage 2a. Moreover, the precision values for stage 2a and stage 2b were 87% and 85%, respectively. The recall values for stage 2a and stage 2b were 90% and 82%, respectively. The F1-score values for stage 2a and stage 2b were 88% and 84%, respectively.

The accuracy of the kNN algorithm on the test dataset was 84%. Among the signals in stage 2a, 90.53% of them were correctly classified, while 9.47% of them were misclassified as signals in stage 2b. Among the signals in stage 2b, 76.29% of them were correctly classified, while 23.71% of them were misclassified as signals in stage 2a. The precision values for stage 2a and stage 2b were 84% and 86%, respectively. The recall values for stage 2a and stage 2b were 91% and 76%, respectively. The F1-score values for stage 2a and stage 2b were 87% and 81%, respectively.

The accuracy of the SVM classification model on the test dataset was 87%. When given 887 stage 2a and 658 stage 2b signals, the model accurately classified 811 signals (91.43%) as from stage 2a and 532 (80.85%) signals from stage 2b. In total, 1343 signals were classified into the correct stage. The precision values for stage 2a and stage 2b were 87% and 88%, respectively. The recall values for stage 2a and stage 2b were 91% and 81%, respectively. The F1-score values for stage 2a and stage 2b were 89% and 84%, respectively.

Overall, the machine learning algorithms built in this study showed good performance in identifying the sub-stage of the AE signal. Among the algorithms, SVM had the highest classification accuracy among the three classifiers (87%), followed by the MLP classifier (86%) and finally the kNN classifier (84%). It should be noted that the accuracy of the classification will be higher with model optimization. Using these built classifiers, the sub-stage of FCG can be determined without knowing AE history and the complex calculation of ΔK. Moreover, the classifiers can be used and trained with more structures under varying environmental conditions. Thus, the classification of fatigue sub-stages using machine learning algorithms is a feasible approach to assess a material’s fatigue-damage state. It is worth pointing out that the proposed method can be potentially applied to a wide range of materials and structure forms. Future studies will focus on expanding the scope of this research by applying the method tested here to a larger and more diverse set of specimens with different structure forms. The optimal measuring points for other structural forms also need to be investigated, and some of latest references available for an optimal layout of sensors are [53,54]. 

## 4. Conclusions

The AE characteristics of Hadfield steel during stable FCG processes were investigated in this study. The AE signals generated from the entire fatigue-load process were acquired and correlated with fatigue-damage evolution. For the first time, machine learning algorithms were used to classify between fatigue sub-stages, and the results clearly demonstrated the effectiveness of classification of fatigue sub-stages using machine learning algorithms. The conclusions obtained from the present study can be drawn as follows:

- Stable and unstable crack growth can be clearly identified based on time–history and the cumulative trend of AE activity.
- The transition from stage 2a to stage 2b in the Paris region is accompanied by a change of slope in AE cumulative count and a drop in count rate vs. ΔK plots, which it is not feasible to observe from da/dN vs. ΔK plots.
- Two main source mechanisms were identified based on waveform characteristics and bispectrum analysis: namely, crack growth and crack opening/closing.
- Machine learning classifiers were shown to be effective in identifying two sub-stages of FCG. SVM has the highest classification accuracy among the three classifiers (87%), followed by the MLP classifier (86%), and finally the kNN classifier (84%).

The results and conclusions presented above clearly indicate that classification of fatigue sub-stages using machine learning algorithms is a feasible approach to assess a material’s fatigue-damage state. For in-service structures, acoustic emission sensors should be placed in areas where NDT or other traditional methods have detected fatigue cracks. The system could acquire AE signals continuously or at intervals, depending on the operational conditions of the structures. Fatigue-damage condition can then be assessed using machine learning algorithms to automatically classify the acquired AE signals, which can help engineers to optimize maintenance and renewal planning. Future studies will aim to broaden the scope of this research by applying the method tested here to a larger and more diverse set of specimens with different structure forms. This expansion will allow for a comprehensive validation of the method and a deeper understanding of its applicability in diverse scenarios.

## Figures and Tables

**Table 1:** Mechanical properties of Hadfield steel [29].

Brinell Hardness/HB	Tensile Strength/MPa	Yield Strength/MPa	Elongation at Break
200	880	320	20%

**Table 2:** Parameters of R50a sensor.

Diameter/mm	Height/mm	Weight/g	Frequency Range/kHz	Temperature/°C
19	22.4	32	150–700	−65~175

**Table 3:** AE features used to train the algorithms.

Feature	Description
Amplitude	The maximum voltage in the acquired AE waveform
Absolute energy	The absolute measure of the electrical energy measured for the AE waveform
Bispectral-based features	Maximum singular value, average singular value, variance of singular value
Count	The number of times the signal rises and exceeds the preset threshold
Crest factor	The ratio of peak amplitude to RMS (root mean square)
Duration	The time interval during which the voltage is above the threshold
Form factor	The ratio of the RMS (root mean square) value to the mean value
Frequency centroid	The frequency where the areas of the frequency spectrum below and above it are the same
Impulse factor	The ratio between a signal’s peak and its mean value
Margin factor	The ratio between a signal’s maximum absolute value and the mean of square root of signal’s absolute value
Peak frequency	The frequency corresponding to the sinusoidal signal with the maximum peak
Risetime	The time period between the first threshold crossing and the signal peak

**Table 4:** Fitting values of crack growth and AE constants for Hadfield steel.

	Crack-Growth Rate (da/dN)			Count Rate (dC/dN)		
Specimen No.	m	C	R-Squared	*p*	B	R-Squared
1	4.7959	8 × 10^−14^	0.9272	0.1358	5.0565	0.0431
2	4.5751	1× 10^−13^	0.9281	0.4135	4.7739	0.0329

**Table 5:** Classification metrics of the MLP classifier.

Sub-Stage	Precision (%)	Recall (%)	F1-Score (%)	Accuracy (%)
Stage 2a	87	90	88	86
Stage 2b	85	82	84	

**Table 6:** Classification metrics of the kNN classifier.

Sub-Stage	Precision (%)	Recall (%)	F1-Score (%)	Accuracy (%)
Stage 2a	84	91	87	84
Stage 2b	86	76	81	

**Table 7:** Classification metrics of the SVM classifier.

Sub-Stage	Precision (%)	Recall (%)	F1-Score (%)	Accuracy (%)
Stage 2a	87	91	89	87
Stage 2b	88	81	84	

## Data Availability

Data is contained within the article.

## Nomenclature

SHM	Structure-health monitoring
AE	Acoustic emission
DC	Direct current
FCG	Fatigue-crack growth
NDT	Non-destructive testing
FCGR	Fatigue-crack growth rate
CNN	Convolutional neural network
CWT	Choi–Williams transform
DCPD	Direct Current Potential Drop
PAC	Physical Acoustic Corporation
DAQ	Data acquisition
MLP	Multilayer perceptron
kNN	k-nearest neighbor
SVM	Support vector machine
RBF	Radial Basis function
SVD	Singular value decomposition
RMS	Root mean square
SFE	Stacking-fault energy
PICC	Plasticity-induced crack closure
ΔK	Stress-intensity factor range
$∆K_{0}$	Stress-intensity factor range at the transition between fatigue sub-stages
da/dN	Crack-growth rate
dC/dN	Acoustic emission count rate
$W_{ij}$	Weight vector
y	Output of the neuron
φ	Activation function
$\varepsilon_{i}$	Input from the i-th neuron
B	Bias
$x_{k}$	Feature vector
$f\left(x\right)$	Decision function
$w^{T}$	Transpose of the weight vector
φ	Kernel function
$y_{k}$	Class label
λ	Penalty parameter
$\xi_{k}$	Slack variable
A	Bispectrum matrix
U	Left singular value matrix
∑	Diagonal matrix of singular values
$V^{T}$	Transpose of the right singular vectors matrix
C, m, B, P	Material constants
R	Stress ratio
k, j	Time lags
$C_{3x}(k,j)$	Third-order cumulant
$B\left(f_{1},f_{2}\right)$	Bispectrum at frequencies $f_{1}$ and $f_{2}$
